# Exploring the molecular mechanism of hepatitis virus inducing hepatocellular carcinoma by microarray data and immune infiltrates analysis

**DOI:** 10.3389/fimmu.2022.1032819

**Published:** 2022-11-11

**Authors:** Yong-Zheng Zhang, Amir Zeb, Lu-Feng Cheng

**Affiliations:** Department of Pharmacology, School of Pharmacy, Xinjiang Medical University, Urumqi, China

**Keywords:** hepatitis virus, hepatocellular carcinoma, immune infiltration, viral carcinogenicity, macrophage polarization, computer simulation

## Abstract

The number of new cases of hepatocellular carcinoma (HCC) worldwide reached 910,000, ranking the sixth, 80% HCC is associated with viruses, so exploring the molecular mechanism of viral carcinogenicity is imperative. The study showed that both HBV and HCV associated HCC and non-viral HCC have the same molecular phenotype (low gene expression and inhibition of immune pathways), but in the tumor immune micro-environment, there is excessive M2-type macrophage polarization in virus-associated hepatocellular carcinoma. To address this phenomenon, the data sets were analyzed and identified five hub genes (POLR2A, POLR2B, RPL5, RPS6, RPL23A) involved in viral gene expression and associated with PI3K-Akt-mTOR pathway activation by six algorithms. In addition, numerous studies have reported that M2-type macrophages participate in the hepatic fibro-pathological process of the development of HCC and are regulated by the PI3K-Akt-mTOR pathway. On this basis, the study showed that hepatitis virus causes abnormal expression of hub genes, leading to the activation of the pathway, which in turn promote the differentiation of M2-type macrophages and eventually promote the formation of liver fibrosis, leading to the occurrence of HCC. In addition, these hub genes are regulated by transcription factors and m^6^A enzyme, and have good prognosis and diagnostic value. With regard to drug reuse, the results suggest that patients with virus-related HCC for whom Cytidine triphosphate disodium salt and Guanosine-5’-Triphosphate are used as supplementary therapy, and may have a better prognosis. In conclusion, the study has identified novel molecules that are carcinogenic to hepatitis viruses and are expected to serve as molecular markers and targets for diagnosis and treatment.

## Introduction

Hepatic malignant tumors can be divided into two major categories: primary and secondary. Primary hepatocellular carcinoma is one of the common malignant tumors in China with a high mortality rate, ranking third in the sequence of deaths from malignant tumors after stomach and esophagus (1). Hepatocellular carcinoma (HCC) is the major subtype of primary hepatocellular carcinoma. In 2020, there were more than 910,000 new HCC cases and 830,000 deaths, which has become a serious public health problem (2). The latest research results have shown that the occurrence of HCC is mainly related to hepatitis B virus (HBV) and hepatitis C virus (HCV) infection (3). At the same time, excessive drinking and smoking are also related to the occurrence of HCC (4).

Systematic understanding of the virus involvement in the occurrence, development and metastasis of HCC is conducive to the early diagnosis and accurate treatment of patients. In theory, the persistent inflammation of hepatocytes caused by viral infection promotes the formation of hepatic fibrosis, which is the basis for the development of HCC (5). Chronic infection caused by HBV to the human body, and chronic hepatitis B, compensated cirrhosis, and decompensated cirrhosis to the onset of HCC are the main pathways for the development of HBV-associated HCC (HBV-HCC) (6). It has been found that the HBx protein carried by HBV can regulate the PI3K-Akt pathway of host hepatocytes, and then activate and release of excessive TGF-β to participate in the occurrence of liver fibrosis (7). TGF-β is a key cytokine involved in fibrogenesis and can be specifically activated by PI3K/Akt (8), which may be an important factor in the induction of HCC by HBV and HCV (9, 10). In addition, studies have found that abnormal PI3K/AKT/mTOR signaling pathway is closely related to HCC resistance (11). Some studies have demonstrated that N6-methyladenylate (m^6^A) modification is involved in the progression of hepatitis B virus-related liver fibrosis by regulating the infiltration of immune cells (12), and at the same time, HBx carried by HBV can interact with the methylase METTL3, which is closely related to the development of HCC (13).

Despite the deepening understanding of the etiology of HCC, the available diagnosis and treatment plan has little effect. Microarray sequencing technology has been applied to genome detection for in-depth exploration of the viral carcinogenicity and tumor development mechanism. However, results from single microarray or low sample size analyses are difficult to gain more recognition. In this study, a prospective method was used that differed from conventional experiments, the expression profiles of HCC (GSE87630), HBV-HCC (GSE55092), and HCV-HCC (GSE19665) from the GEO database were integrated. Differentially expressed genes (DEGs) were identified in HCC, HBV-HCC, and HCV-HCC, compared to normal liver tissue. DEG was analyzed for gene set enrichment using “h.all. v7.4” and in addition, three types of HCC were analyzed for tumor immune invasion using CIBERSORT. To further analyze the molecular mechanisms of the involvement of the two hepatitis viruses in the development of HCC, an ExpressAnalyst was used to combine the three data sets. Compare with HCC, and in contrast to HCC, the DEG of HBV-HCC and HCV-HCC were identified, and two co-homology genes were crossed to explore the molecular basis of viral involvement in HCC. Six sub-networks of key molecular interactions were obtained using the MCODE method, and gene ontology (GO) and Kyoto Encyclopedia of Genes and Genomes (KEGG), analyses were performed using the DAVID database. In addition, the hub genes were obtained by analyzing the sub-networks using six algorithms, and the correlation was explored between those genes and immune-infiltrating cells using Pearson correlation analysis. A separate cohort (GSE121248 and GSE69715) was integrated with HCC (GSE87630) to verify the stability of hub gene expression. Meanwhile, the protein levels of those genes were verified in the human protein map (HPA). The prognostic value of hub genes was verified on the UALCAN data analysis platform, and the receiver operating characteristic (ROC) curve was used to analyze the diagnostic value of hub genes in distinguishing HCC tissues (HBV-HCC and HCV-HCC) from normal liver tissues. Transcription factor and m^6^A methylation predictions were performed using hTFtarget and m6A2Target and validated in microarrays for hub gene. Therapeutic drugs related to genes were explored and developed by STITCH, and effect intensity analysis was performed on computer simulation software (Schrodinger). The aim of this study is to provide new insights into the pathogenesis of viral involvement in HCC and to identify new prognostic markers and precise drug targets through comprehensive analysis.

## Methods

### Data extraction, processing and consolidation

All data sets (**Table 1**) were from GEO (https://www.ncbi.nlm.nih.gov/geo) database, were log transformed and normalized. The differentially expressed genes (DEGs) between hepatocellular carcinoma (HCC) tissues and normal liver tissues were screened out by using “limma” function. The screening criteria for DEGs were adjusted P value < 0.05 and LogFC≥1. Volcanic map of gene distribution by using that ggplot2 function. Batch effects were removed from the dataset using “combat” function by the online platform ExpressAnalyst (https://dev.expressanalyst.ca/ExpressAnalyst/).

**Table 1:** The characteristics of the datasets used in the present study.

Accession No.	Tissue sources	Platform	Samples(Control: Case)	Data annotation
GSE87630	Liver	GPL6947	30:64	HCC
GSE55092	Liver	GPL570	81:39	HBV-HCC
GSE121248	Liver	GPL570	37:70	HBV-HCC
GSE19665	Liver	GPL570	5:5	HCV-HCC
GSE69715	Liver	GPL570	66:37	HCV-HCC

### GSEA and immune infiltration analysis

To clarify the gene effects caused by DEGs of HCC, the R package “GSEA” was utilized to obtain the GSEA enrichment scores of hallmark pathways (h.all.v7.4.entrez) (14). HCC immune infiltration analysis was performed on the dataset using the “CIBERSORT” method on the Sangerbox3.0 platform (http://vip.sangerbox.com). Due to the small sample size of GSE19665 for HCV-HCC and the potential for large deviations in analysis results, an external independent dataset, GSE69715, was selected for immune invasion analysis.

### Analysis of protein-protein interaction network and functional enrichment

Interaction between intersecting gene were analyzed using a STRING database (15). The MCODE functional module was used to cluster the genes and construct a gene sub-network in cytoscape (version 3.8.3). The DAVID 2021 was used for analyzing GO and KEGG pathway of module gene (16). P<0.05 is considered to be statistically significant.

### Gene screening and co-expression network construction

In order to identify the hub genes in the common genes, the six algorithms (Closeness, Stress, EPC, Degree, MNC, and Radiality) of the “cytohubba” module were used for gene sequencing, and the intersection analysis of the top 30 hub genes was performed using the R software “Upset” package. Gene co-expression analysis and functional enrichment analysis of the common hub genes were performed using the GeneMania platform (17).

### Correlation analysis of gene expression

To explore the association between hub genes and M2 macrophage infiltration, Pearson’s method was used for gene association analysis on HBV-HCC and HCV-HCC data sets. Due to the small sample size of GSE19665 for HCV-HCC and the potential for large deviations in analysis results, an external independent dataset, GSE69715, was selected for gene correlation analysis.

### Analysis of mRNA and protein expression

Expression data of hub genes in HCC with different stages and HCC with TP53 mutation were obtained from UALCAN (18), which is a platform for processing data from TCGA. Extraction of protein levels of viral carcinogenic hub genes in HCC Tissue and Normal Liver Tissue from the Human Protein Atlas (HPA) (19). The staining intensity was divided into strong, medium, weak or negative. Expression levels included high, medium, low, and none detected. The proportion of stained cells was three-tiered (> 75%, 25-75%, or < 25%).

### Survival analysis and ROC curve drawing

The survival analysis was analyzed by using the UALCAN. Survival analysis was performed by Kaplan-Meier and log-rank test. The expression levels of hub genes were applied for ROC analysis to estimate their diagnostic significance to distinguish between HCC and normal in two independent external sets (GSE121248 and GSE69715) and internal sets (GSE55092 and GSE19665). The area under curve (AUC) > 0.5 was considered to have diagnostic value.

### Transcriptional factor and m^6^A enzyme prediction analysis

In order to find the molecules that regulate the hub genes, hTFtarget database was used to predict the TFs of the hub genes and validated the expression levels in the data set (20). Because of the extensive presence of m^6^A enzyme modification after RNA transcription, m^6^A enzyme prediction (21) and validation on the hub genes were performed, and constructed a network diagram based on the interaction relationship.

### Drug screening based on hub genes

The sequence of hub gene was obtained from NCBI and homology modeling was performed using Swiss Mode (22). Schrodinger Glide and IFD modules were used to molecular dock the hub gene structure with the best score with drug molecules. The lower binding energy of drug to target (hub gene) indicates more stable binding. Docking mode diagrams are drawn using LigPlus (23).

## Results

### DGE identification and GSEA in HCC

**Figure 1:**
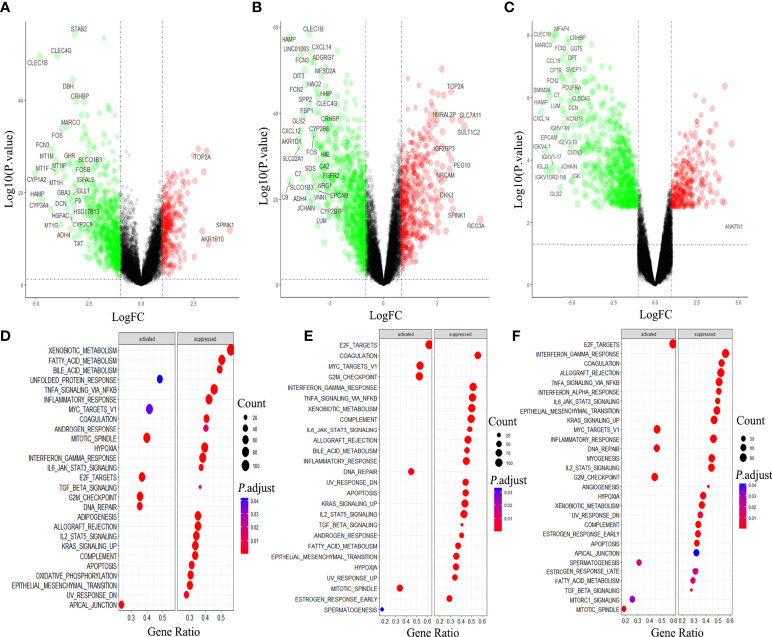
Differential gene analysis and GSEA analysis in hepatocellular carcinoma (HCC). Gene expression and GSEA analysis of HCC **(A, D)**, HBV-HCC **(B, E)** and HCV-HCC **(C, F)** by using R 3.6.3.

We compared the gene expression in Hepatocellular carcinoma (HCC) with that in the normal tissue, and obtained 1158 DEGs, among which 819 genes were down-regulated, accounting for more than 70% (**Figure 1A**). In HBV-HCC and HCV-HCC, the down-regulated genes account for 63% and 83% of DEG, respectively (**Figures 1B, C**). Downstream analysis of DGE using GSEA revealed that virus associated HCC was similar to HCC, mainly manifested are the activation of DNA damage and G2M checkpoint, and inhibition of hypoxia, apoptosis and immune response (**Figures 1D-F**).

### Immune infiltration analysis in HCC

**Figure 2:**
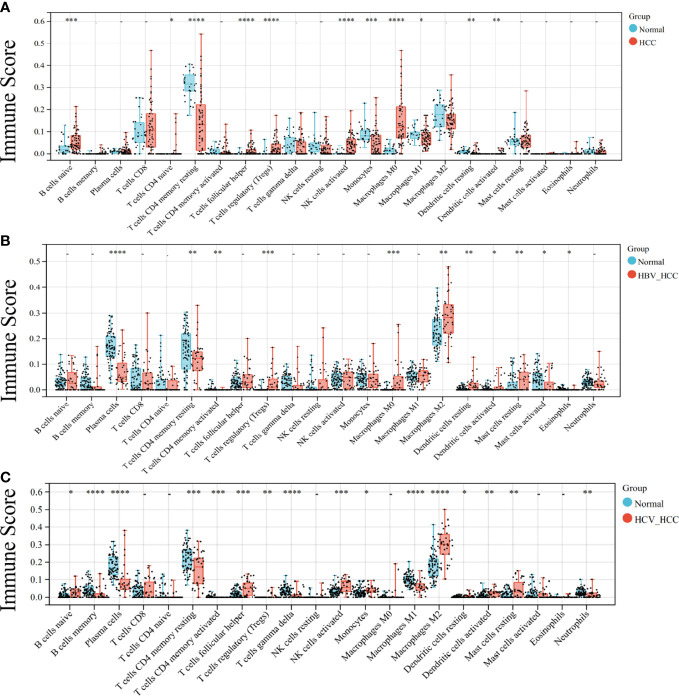
Analysis of immune infiltration in HCC. Immune infiltration of HCC **(A)**, HBC-HCC **(B)**, and HCV-HCC **(C)** using CIBERSORT. Statistical significance is noted with asterisk: * < 0.05, ** < 0.01, *** <0.001, ****< 0.0001.

As both the virus-induced HCC and the HCC developed immunologic derangement, we performed immune infiltration analysis on them to distinguish the immune cell levels. The results showed that CD4 T cells and Treg cells were the common manifestations of them (**Figures 2A-C**). Interestingly, M2-type macrophages and plasma cells proliferate extensively in virus-induced HCC and cause infiltration of mast cells resting in HBV-HCC, but are not observed in HCC.

### Genetic differences between virus-induced HCC and HCC

**Figure 3:**
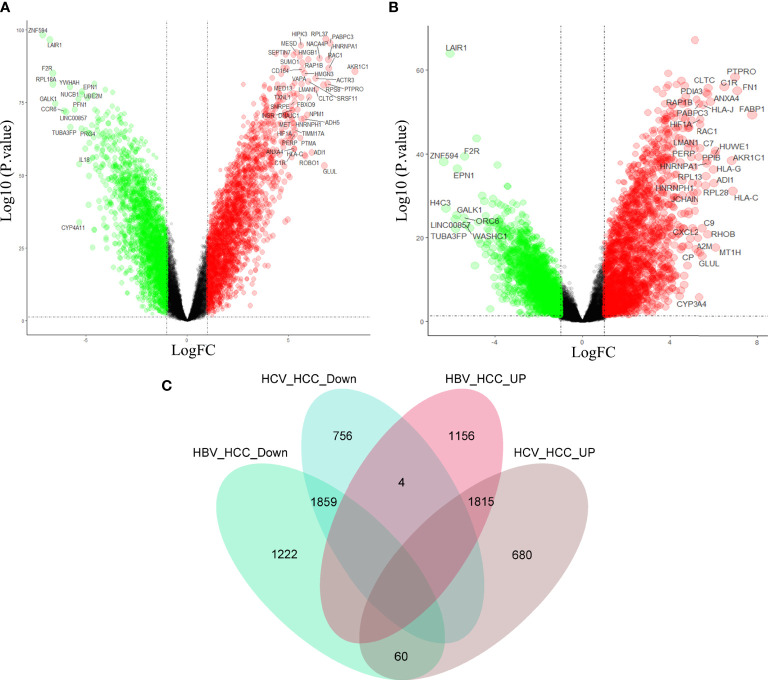
Differential gene analysis and intersection gene Venn diagram. Gene expression profiles of HBV-HCC **(A)** and HCV-HCC **(B)** compared with HCC. **(C)** Drawing the intersecting Venn diagram to clarify the molecular mechanism of HCC co-caused by hepatitis viruses.

In order to further clarify the molecular mechanism of virus participation in HCC, the above three data sets were combined in the study. See **Figure S1** for quality control. Differential gene analyses of HBV-HCC and HCC, HCV-HCC and HCC, revealed that compared with HCC, HBV-HCC had 3,141 down-regulated genes and 2,975 up-regulated genes (**Figure 3A**), and HCV-HCC had 2,619 down-regulated genes and 2,555 up-regulated genes (**Figure 3B**). In order to find the possible molecular phenotype of HCC caused by the combination of the two viruses, Venn mapping of all up-regulated and down-regulated genes was conducted, and was found that 1859 down-regulated genes and 1815 up-regulated genes participated in the development of HCC (**Figure 3C**). The difference between HCC and virus-associated HCC is that the two expression patterns differ significantly, and this expression pattern may be related to viral carcinogenicity.

### Protein-protein interaction analysis and enrichment analyses

**Figure 4:**
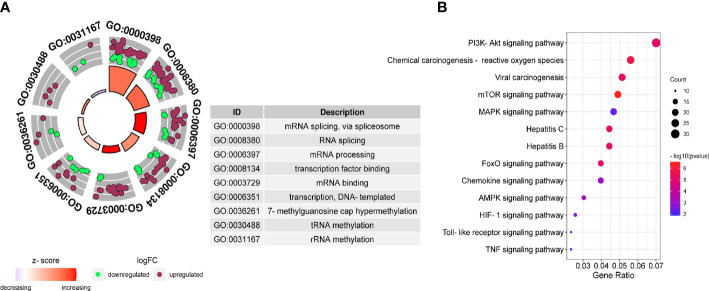
Gene Ontology (GO) and Kyoto Encyclopedia of Genes and Genomes (KEGG) pathway of DEGs. GO **(A)** and KEGG **(B)** enrichment analysis were performed on the key module genes.

Further analysis of the molecular patterns of two hepatitis viruses participating in HCC. We performed protein interaction analysis and MCODE analysis on the intersecting genes. Due to the limitation of the database on the number of genes, a interaction analysis was conducted on the up-regulated and down-regulated genes respectively and the first three molecular networks of down-regulated genes with the scores of 35.925, 14.615 and 12.414 (**Figure S2A**), and the first three networks of up-regulated genes with the scores of 13.762, 12.557 and 10.618 (**Figure S2B**) were obtained. Considering the molecular cascade effect, combining the genes of the above six sub-networks and conducting GO and KEGG analysis, it is clear that the modular gene is related to the methylation activation of mRNA splicing, mRNA processing and tRNA methylation (**Figure 4A**), and in addition, it is also involved in the PI3K-Akt pathway, viral carcinogenesis, mTOR signaling pathway, hepatitis B, and hepatitis C (**Figure 4B**).

### Hub genes screening and co-expression network construction

**Figure 5:**
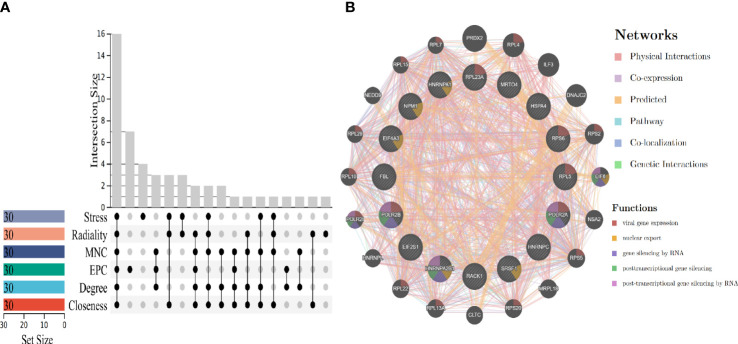
Hub genes screening and gene co-expression analysis. **(A)** Hub genes screening by using six algorithms. **(B)** Hub genes co-expression and functional enrichment analysis.

To obtain biomarkers or drug targets involving carcinogenesis of hepatitis virus, the study identified 16 potential hub genes using six algorithms (**Figure 5A**), The related functions and co-expression network of those genes were analyzed on the basis of the database. It shows the complex network with the physical interactions of 34.30%, co-expression of 32.68%, predicted of 24.59%, and co-localization of 2.73% (**Figure 5B**). It is noteworthy that, ribosomal protein L23A (RPL23A), ribosomal protein S6 (RPS6), ribosomal protein L5 (RPL5), RNA polymerase II subunit A (POLR2A) and RNA polymerase II subunit B (POLR2B) participated in viral gene expression. In addition, POLR2A, POLR2B and heterogeneous nuclear ribonucleoprotein A2/B1 (HNRNPA2B1) are related to post-transcriptional gene silencing by RNA.

### Correlation analysis between hub genes and immune pathway

**Figure 6:**
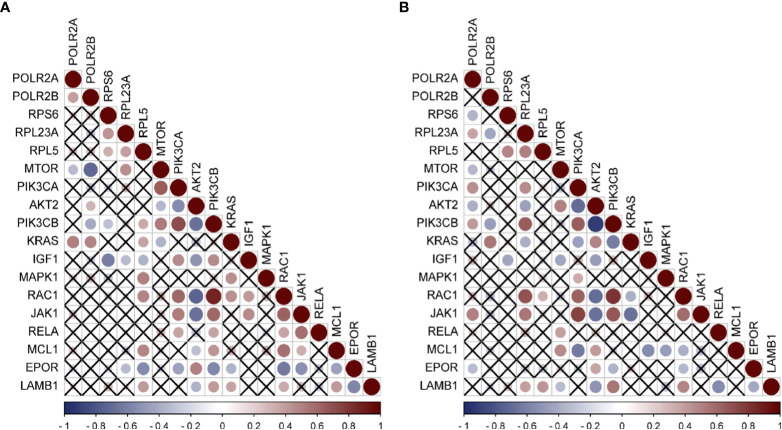
Expression correlation analysis of hub genes and Immune related genes **(A)** Correlation analysis of HBV-HCC immune pathway related genes and hub genes by using GSE55092. **(B)** Correlation analysis of HCV-HCC immune pathway related genes and hub genes by using GSE69715. Red was positively correlated and blue was negatively correlated. The size of the circle represented the magnitude of correlation, and the cross indicated no correlation.

Since a large number of M2-type macrophages infiltrate HCC caused by HBV and HCV, and the activation of PI3K-Akt-mTOR pathway is related to the M2 polarization of macrophages (11, 24), Through literature review, it was found that 13 differential genes (25–36), including mTOR, PIK3CA, AKT2, and PIK3CB, involved in the M2-type polarization of macrophages, according to the genes related to M2-type polarization. We first analyzed the expression of these 13 genes in HBV-HCC and HCV-HCC, and found that mTOR was down-regulated while other genes were up-regulated (**Figure S3**), which was consistent with the molecular characteristics of macrophage polarization. Furthermore, the analysis of the correlation between hub genes and these genes was carried out, and the results emphasized that in the HBV-HCC data set, RPL5 had a positive correlation with the expression of six genes including PIK3CB, KRAS, and MAPK1 (**Figure 6A**). However, the expressions of POLR2A and POLR2B were negatively correlated with mTOR expression, but positively correlated with KRAS expression. In HCV-HCC, RPL23A is positively correlated with RAC1, JAK1, PIK3CD, etc. (**Figure 6B**). Similarly, PIK3CB is positively correlated with the expression of POL2A, but negatively correlated with PLOR2B. The regulation of PIK3CB by hub genes may be an important reason for the polarization of M2-type macrophages.

### mRNA and protein expression verification of hub genes

**Figure 7:**
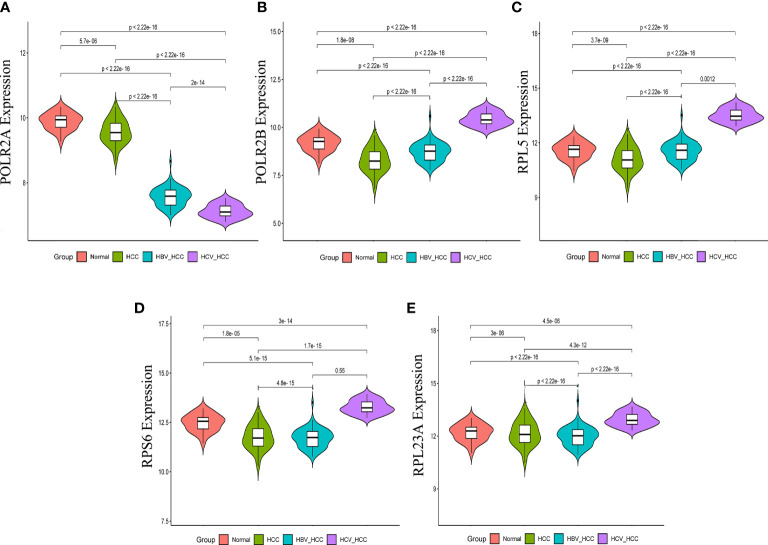
Expression of hub genes in HCC, HBV-HCC and HCV-HCC liver tissues. The expression data of POLR2A **(A)**, POLR2 **(B)**, RPL5 **(C)**, RPS6 **(D)** and RPL23A **(E)** in different groups were analyzed by t-test.

**Figure 8:**
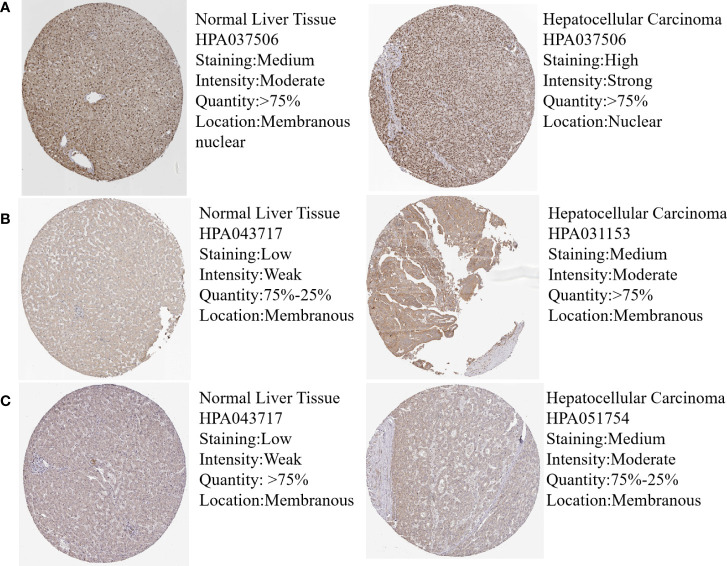
Protein expression of hub genes in normal and HCC liver tissues. Protein levels of POLR2B **(A)**, RPS6 **(B)** and RPL23A **(C)** in normal liver tissue and HCC liver tissue.

In order to validate the hub gene-expression stability, independent queues that contained HBV-HCC and HCV-HCC were selected for the analysis of these hub genes expression levels. The results demonstrated that while comparing with HCC, the POLR2A was significantly down-regulated, however POLR2B, RPL5, RPS6 and RPL23A were significantly up-regulated in HBV-HCC and HCV-HCC (**Figure 7**). Moreover, the relevance between hub genes and clinical characteristics (cancer stage and TP53 mutation) in HCC patients was analyzed using the UALCAN. Higher mRNA expression of gene is associated with staging in patients with HCC (**Figure S4**). Similarly, the mRNA expression levels of hub genes were higher in HCC with TP53 mutation. Moreover, the protein expression levels of POLR2B, RPS6 and RPL23A in HCC tissue were higher than those in normal liver tissue by observing the immunohistochemistry results (**Figure 8**).

### Prognostic analysis and diagnostic value of hub genes

**Figure 9:**
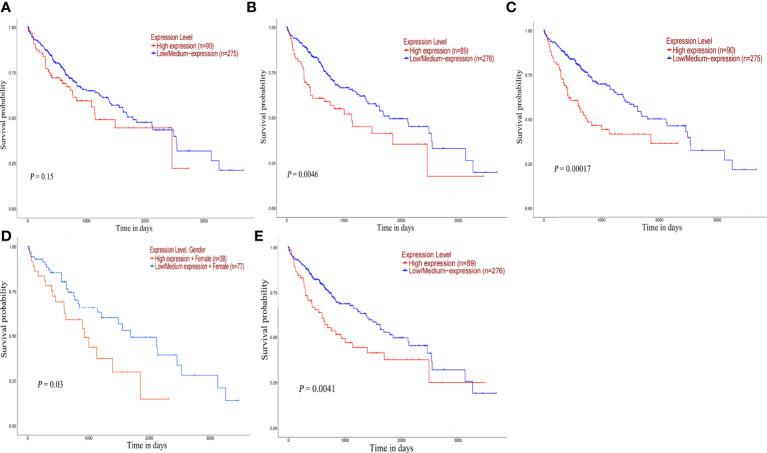
Overall survival analysis of hub genes. Survival curve for POLR2A **(A)**, POLR2B **(B)**, RPL5 **(C)**, RPS6 **(D)** and RPL23A **(E)** in HCC patients from TCGA database. The expression data were analyzed using log-rank test.

**Figure 10:**
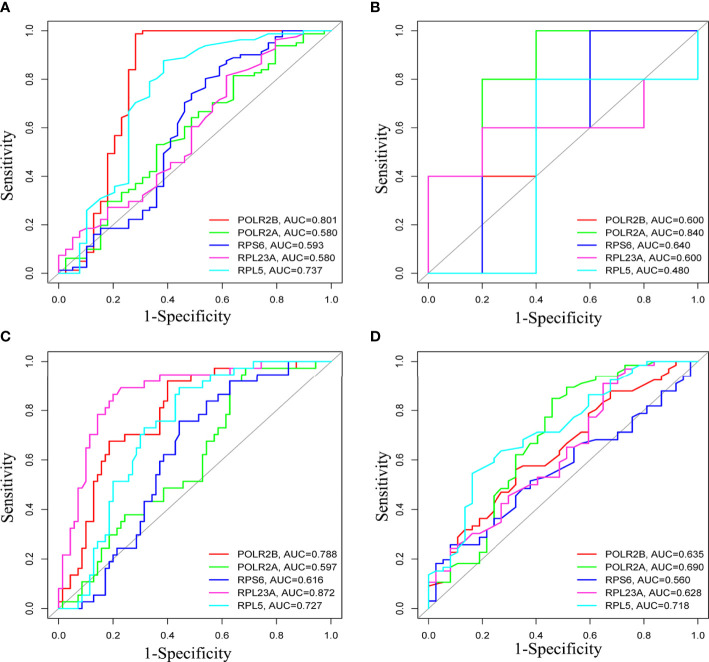
ROCcurve analysis to assess the diagnostic value of hub genes in differentiating HBV-HCC or HCV-HCC from liver tissues. **(A, B)** were HBV-HCC and HCV-HCC, respectively, in the internal data set, **(C, D)** were HBV-HCC and HCV-HCC, respectively, in the external verification set.

The survival analysis of hub gene was carried out by TCGA data set (HCC) in UALCAN. Although POLR2A expression was not associated with patient survival rate (**Figure 9A**), the results reveled that high expression of POLR2B (P <0.01), RPL5 (P < 0.001), RPS6 (P < 0.05), and RPL23A (P < 0.01) were concerned with shorter survival rates (**Figures 9B-E**). In summary, POLR2B, RPL5, RPS6, and RPL23A may be used as biomarkers to estimate the prognosis of HCC patients. To determine the diagnostic significance of these genes in virus-induced HCC and normal, ROC analysis was performed using data from internal sets and AUC values for all five genes were greater than 0.5 (**Figures 10A, B**). In general, the diagnostic value of viral oncogenes in HBV-HCC is higher than that in HCV-HCC. The same results appeared in separate external data sets (**Figures 10C, D**). Therefore, these genes have value for further development as diagnostic biomarkers in virus-related HCC.

### Prediction and validation of transcription factor and m^6^A enzymes

**Figure 11:**
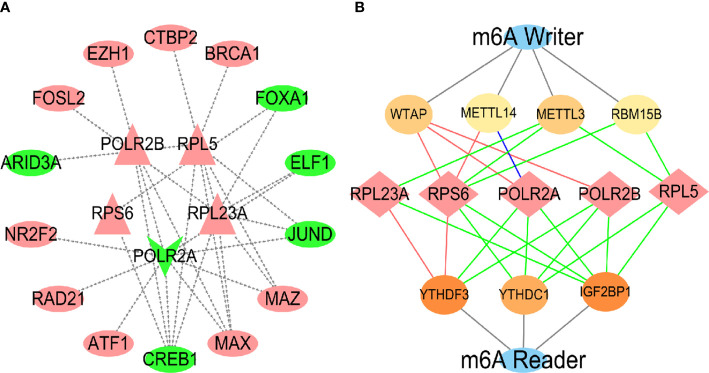
Prediction and validation of TF and m^6^A enzymes. In the TFs regulatory network **(A)**, the inside is the central gene, and the outside is the transcription factor. Red represents up-regulation, and green represents down-regulation. In the m^6^A regulatory nerwork **(B)**, the red lines represented protein-protein interactions, the green lines represented protein-RNA interactions, and the blue lines represented protein DNA interactions.

As the high expression of hub genes is involved in the development of HCC caused by virus, we further explored the molecular mechanism for regulating the hub genes. Transcription factor (TF) prediction has revealed that a variety of TF are involved in the regulation of hub genes expression (**Figure 11A**), and it was identified in the internal data set that high expression of 9 TFs and low expression of 5 TFs may be important causes of abnormal changes in hub genes (**Figure S5**). Because the module gene is closely related to RNA methylation (**Figure 4A**), the hub genes was predicted by m^6^A methylation and verified by internal data sets. The results showed that the hub genes were regulated by m^6^A writer and reader proteins (**Figures 11B**, **S6**). Analyzing the prognosis of m^6^A, identified that the high expression of mRNA-splicing regulator WTAP (WTAP), Putative RNA-binding protein 15B (RBM15B) and N6-adenosine-methyltransferase catalytic subunit (METTL3) were related to the survival time of HCC patients (**Figure S7**).

### Drug screening and computer simulation analysis of hub genes

**Figure 12:**
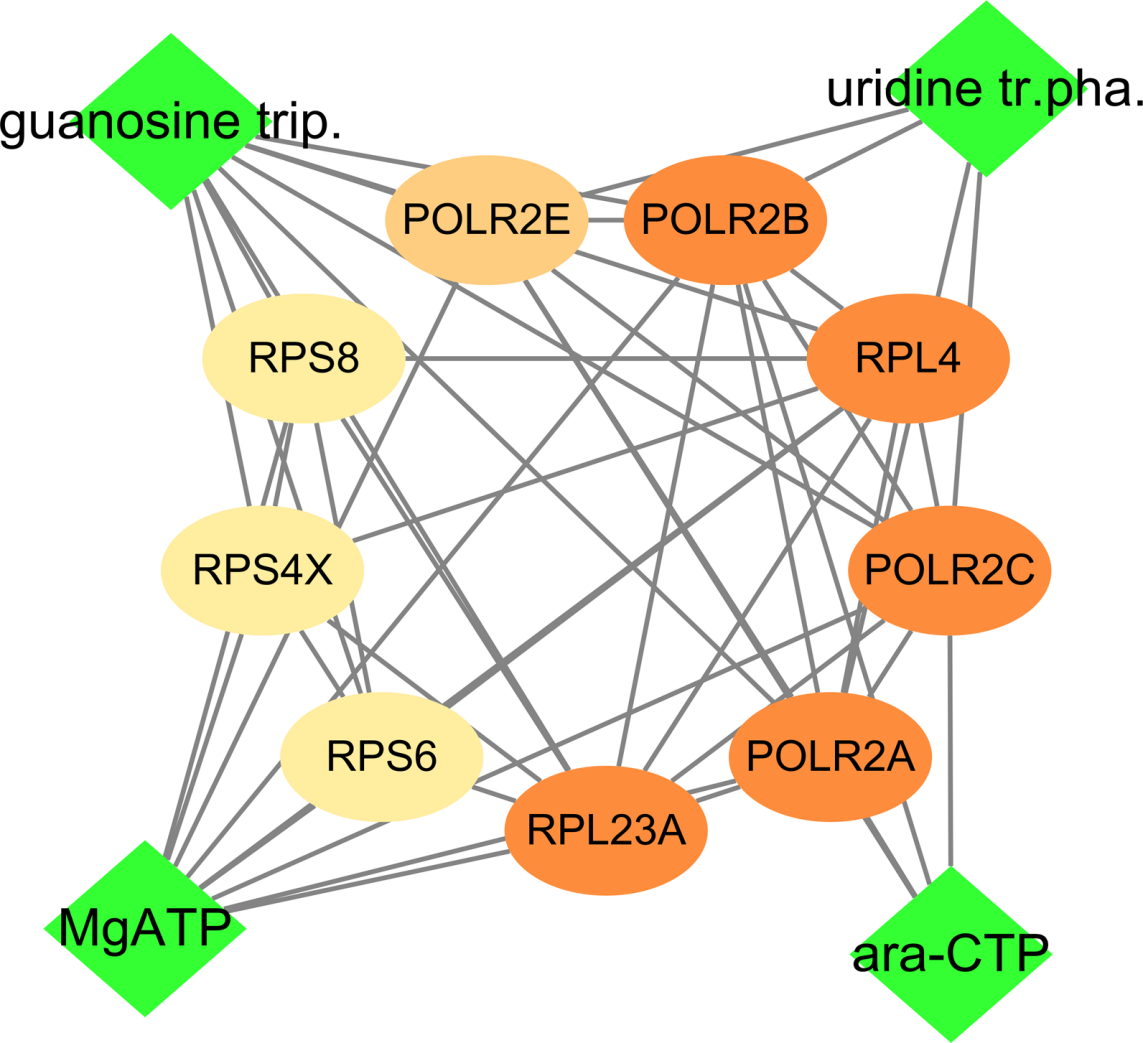
Drug-hub gene interacvtion. Green rhombus is medicine, and ellipse is gene including POLR2B, RPS6 and RPL23A.

As the high mRNA and protein expressions of POLR2B, RPS6, and RPL23A in HCC tissues and participate in the expression of viral genes, which is closely related to the prognosis and diagnosis of patients, compound-protein interaction analysis was conducted (**Figure 12**). Cytidine triphosphate disodium salt (ara-CTP) and Guanosine-5’-Triphosphate (guanosine trip) have the ability to inhibit hepatitis virus replication (37, 38). A semi-flexible (Glide) and induced-fit docking (IFD) methods were used in the study, and it was found that the two drugs had low binding energy to the hub genes and more hydrogen bond interactions (**Table 2**, **Figure S8**).

**Table 2:** docking score of drug and hub genes.

Hub genes	ara-CTP (kCal·mol^-1^)	guanosine trip (kCal·mol^-1^)
POLR2B Glide/IFD	-6.78/-7.27	-6.94/-8.005
RPS6 Glide/IFD	-8.28/-8.527	-7.21/-9.271
RPL23A Glide/IFD	-6.74/-6.354	-7.87/-7.162

Note: Glide docking is based on geometry and is used to search for possible binding sites of drugs. IFD is based on the “lock-key theory” and is used as a method to evaluate drug affinity.

## Discussion

The carcinogenic effect of virus is obvious, especially virus-related hepatocellular carcinoma (HCC). However, its carcinogenic mechanism still needs to be further clarified. We started from three independent cohorts of HCC and found their differences. Both DNA damage and G2M checkpoint inhibition are caused by the over-expression of HBx protein carried by HBV (39, 40), which is involved in the development of HCC. Therefore, targeted regulation of these two processes may be an important strategy for the treatment of virus-related HCC. The immune response that lymphocytes are widely suppressed is related to the development of HCC (41, 42). Hence the infiltration of two T cell subtypes in the lesion site decrease, was observed. In addition, high-expression M2-type macrophages are highly correlated with tissue fibrosis and tumor immune escape (43). Unlike HCC, virus-related HCC shows a large number of M2-type macrophages infiltration, which may be closely related to the viral carcinogenicity. Further comparison analysis was performed on the difference in molecular expression between virus-associated HCC and HCC, and it was unfolded that the key network genes of virus-associated HCC were mainly involved in the chemical modification of RNA, which is closely related to the poor prognosis of tumor patients (44). These genes are inevitably involved in the activation of immune pathways and the development of hepatitis. It is significant that PI3K-Akt and mTOR pathways are involved in the polarization of M2-type macrophages (24, 45).

To further screen for molecular markers or targets, five hub genes (POLR2A, POLR2B, RPL5, RPS6 and RPL23A) were identified which are involved in viral gene expression using six algorithms. The existing evidence found that these five genes participated in viral gene expression (46–52), while the mechanism involved in liver cancer was not reported. For this reason, the relationship between the hub genes and PI3K-Akt/mTOR pathway was explored. The results showed that the hub genes were positively correlated with the expression of multiple targets in the pathway. A large number of studies have reported that M2-type macrophage infiltration promotes the pathological process of hepatic fibrosis in HCC (53–55). It was found significant that these hub genes caused the M2-type polarization of macrophages by activating the PI3K-Akt-mTOR pathway, and then led to hepatic fibrosis and participated in the development of HCC.

In view of the carcinogenic effect of the hub gene, was further verified by independent cohort from GEO and the results identified that the hub gene POLR2A was low expressed in virus-related HCC, and POL2B, RPL5, RPS6, and RPL23A were high expressed, which were consistent with the expression in the internal data sets. To expand whether the hub gene expression pattern was the same as HCC, high expression of the hub genes mRNA in tumor tissues was found during validation of the HCC data set in the TCGA database. The differential expression of POLR2A may be due to its association with only virus-associated HCC and not with HCC. The HPA database shows that the proteins of POL2B, RPS6 and RPL23A are highly expressed in HCC tissues, and are expected to be molecular targets for anti-tumor. Furthermore, it was unveiled that high expression of hub genes, except POLR2A, was associated with a poor prognosis in patients with HCC. For target-based drug reuse, the ara-CTP and guanosine trip can form multi-hydrogen-bond complexes with POL2B, RPS6 and RPL23A, with low binding energy, suggesting that they may have a better prognosis when used as adjuvant therapy for patients with virus-associated HCC, which is clear from the results. For the regulation of expression of hub genes, it was speculated that abnormal expression of 14 transcription factors associated with high expression of hub genes through database and internal validation, and the m^6^A enzyme may play an important role in post-transcriptional modification. In conclusion, this study identified the novel molecules involved in virus-associated HCC, which can provide a reference for the diagnosis and treatment of patient.

## Data availability statement

Publicly available datasets were analyzed in this study. This data can be found here: https://www.ncbi.nlm.nih.gov/geo/browse.

## Author contributions

Y-ZZ and L-FC performed the study and wrote the manuscript. AZ performed drug screening based on hub genes. All authors contributed to the article and approved the submitted version.

## References

[1] Zheng R Qu C Zhang S Zeng H Sun K Gu X . Liver cancer incidence and mortality in China: temporal trends and projections to 2030. Chin J Cancer Res (2018) 30(6):571–9. doi: 10.21147/j.issn.1000-9604.2018.06.01 PMC6328503 30700925

[2] Sung H Ferlay J Siegel RL Laversanne M Soerjomataram I Jemal A . Global cancer statistics 2020: GLOBOCAN estimates of incidence and mortality worldwide for 36 cancers in 185 countries. CA Cancer J Clin (2021) 71(3):209–49. doi: 10.3322/caac.21660 33538338

[3] GBD 2019 Hepatitis B Collaborators . Global, regional, and national burden of hepatitis b, 1990-2019: a systematic analysis for the global burden of disease study 2019. Lancet Gastroenterol Hepatol (2022) 7(9):796–829. doi: 10.1016/s2468-1253(22)00124-8 35738290 PMC9349325

[4] Baecker A Liu X La Vecchia C Zhang ZF . Worldwide incidence of hepatocellular carcinoma cases attributable to major risk factors. Eur J Cancer Prev (2018) 27(3):205–12. doi: 10.1097/cej.0000000000000428 PMC5876122 29489473

[5] Xie Y . Hepatitis b virus-associated hepatocellular Carcinoma. [J]. Adv Exp Med Biol (2017) 1018:11–21. doi: 10.1007/978-981-10-5765-6_2. 29052129

[6] Lefeuvre C Le GH Ducancelle A . A pleiotropic role of the hepatitis b virus core protein in hepatocarcinogenesis. Int J Mol Sci (2021) 22(24):13651–1. doi: 10.3390/ijms222413651 PMC8707456 34948447

[7] Dong KS Chen Y Yang G Liao ZB Zhang HW Liang HF . TGF-β1 accelerates the hepatitis b virus X-induced malignant transformation of hepatic progenitor cells by upregulating miR-199a-3p. Oncogene. (2020) 39(8):1807–20. doi: 10.1038/s41388-019-1107-9 PMC7033045 31740785

[8] Liu Y Liu H Meyer C Li J Nadalin S Königsrainer A . Transforming growth factor-β (TGF-β)-mediated connective tissue growth factor (CTGF) expression in hepatic stellate cells requires Stat3 signaling activation. J Biol Chem (2013) 288(42):30708–19. doi: 10.1074/jbc.M113.478685 PMC3798541 24005672

[9] Jabłońska J Pawłowski T Laskus T Zalewska M Inglot M Osowska S . The correlation between pretreatment cytokine expression patterns in peripheral blood mononuclear cells with chronic hepatitis c outcome. BMC Infect Dis (2015) 15:556. doi: 10.1186/s12879-015-1305-1 26637466 PMC4670510

[10] Toshikuni N Matsue Y Minato T Hayashi N Tsutsumi M . Association between transforming growth factor-β1 -509 C>T variants and hepatocellular carcinoma susceptibility: a meta-analysis. Neoplasma. (2016) 63(6):961–6. doi: 10.4149/neo_2016_615 27596296

[11] Bamodu OA Chang HL Ong JR Lee WH Yeh CT Tsai JT . Elevated PDK1 expression drives PI3K/AKT/MTOR signaling promotes radiation-resistant and dedifferentiated phenotype of hepatocellular carcinoma. Cells. (2020) 9(3):746. doi: 10.3390/cells9030746 32197467 PMC7140693

[12] Zhao T Qi J Liu T Wu H Zhu Q . N6-methyladenosine modification participates in the progression of hepatitis b virus-related liver fibrosis by regulating immune cell Infiltration. Front Med (2022) 9:821710–0. doi: 10.3389/fmed.2022.821710 PMC8924664 35308519

[13] Geon WK Aleem S . Hepatitis b virus X protein recruits methyltransferases to affect cotranscriptional N6-methyladenosine modification of viral/host RNAs [J]. P Natl A Sci India B (2021) 118(3):e2019455118. doi: 10.1073/pnas.2019455118 PMC7826408 33397803

[14] Hanzelmann S Castelo R Guinney J . Gsva: Gene set variation analysis for microarray and rna-seq data. BMC Bioinf. (2013) 14:7. doi: 10.1186/1471-2105-14-7 PMC3618321 23323831

[15] Szklarczyk D Morris JH Cook H Kuhn M Wyder S Simonovic M . The STRING database in 2017: quality-controlled protein-protein association networks, made broadly accessible. Nucleic Acids Res (2017) 45(D1):D362–8. doi: 10.1093/nar/gkw937 PMC5210637 27924014

[16] Huang DW Sherman BT Lempicki RA . Systematic and integrative analysis of large gene lists using DAVID bioinformatics resources. Nat Protoc (2009) 4:44–57. doi: 10.1038/nprot.2008.211 19131956

[17] Franz M Rodriguez H Lopes C Zuberi K Montojo J Bader GD . GeneMANIA update 2018. Nucleic Acids Res (2018) 46:W60–4. doi: 10.1093/nar/gky311 PMC6030815 29912392

[18] Chandrashekar DS Karthikeyan SK Korla PK Patel H Shovon AR Athar M . UALCAN: An update to the integrated cancer data analysis platform. Neoplasia (2022) 25:18–27. doi: 10.1016/j.neo.2022.01.001 35078134 PMC8788199

[19] Colwill K . Renewable protein binder working Group,Gräslund Susanne,A roadmap to generate renewable protein binders to the human proteome. Nat Methods (2011) 8:551–8. doi: 10.1038/nmeth.1607 21572409

[20] Zhang Q Liu W Zhang HM . hTFtarget: A comprehensive database for regulations of human transcription factors and their targets. Genom Proteom Bioinf (2020) 18:120–8. doi: 10.1038/nmeth.1607 PMC7647694 32858223

[21] Deng S Zhang H Zhu K Li X Ye Y Li R . M6A2Target: a comprehensive database for targets of m^6^A writers, erasers and readers. Brief Bioinform (2021) 22:bbaa055. doi: 10.1093/bib/bbaa055 32392583

[22] Waterhouse A Bertoni M Bienert S Studer G Tauriello G Gumienny R . SWISS-MODEL: homology modelling of protein structures and complexes. Nucleic Acids Res (2018) 2(46):W296–W303. doi: 10.1093/nar/gky427 PMC6030848 29788355

[23] Laskowski RA Swindells MB . LigPlot+: multiple ligand-protein interaction diagrams for drug discovery. J Chem Inf Model (2011) 51:2778–86. doi: 10.1021/ci200227u 21919503

[24] Cai J Huang L Tang H Xu H Wang L Zheng M . Macrophage migration inhibitory factor of thelazia callipaeda induces M2-like macrophage polarization through TLR4-mediated activation of the PI3K-akt pathway. FASEB J (2021) 35(9):e21866–6. doi: 10.1096/fj.202100676R 34416031

[25] Liu X Liu J Xu Y Zhang X Wang YX Qing LH . Membrane metallo-endopeptidase mediates cellular senescence induced by oncogenic PIK3CA accompanied with pro-tumorigenic secretome. Int J Cancer (2019) 145:817–29. doi: 10.1002/ijc.32153 30671946

[26] Wu X Chen H Wang Y Gu Y . Akt2 affects periodontal inflammation *via* altering the M1/M2 ratio. J Dent Res (2020) 99:577–87. doi: 10.1177/0022034520910127 32228353

[27] Lu C Shi W Hu W Zhao Y Zhao X Dong F . Endoplasmic reticulum stress promotes breast cancer cells to release exosomes circ_0001142 and induces M2 polarization of macrophages to regulate tumor progression. Pharmacol Res (2022) 177:106098. doi: 10.1016/j.phrs.2022.106098 35091089

[28] Dai E Han L Liu J Xie Y Kroemer G Klionsky DJ . Autophagy-dependent ferroptosis drives tumor-associated macrophage polarization *via* release and uptake of oncogenic KRAS protein. [J] Autophagy (2020) 16:2069–83. doi: 10.1080/15548627.2020.1714209 PMC7595620 31920150

[29] Spadaro O Camell CD Bosurgi L Nguyen KY Youm YH Rothlin CV . IGF1 shapes macrophage activation in response to immunometabolic challenge. Cell Rep (2017) 19:225–34. doi: 10.1016/j.celrep.2017.03.046 PMC5513500 28402847

[30] Xu W Dong X Ding J Liu JC Xu JJ Tang YH . Nanotubular TiO regulates macrophage M2 polarization and increases macrophage secretion of VEGF to accelerate endothelialization *via* the ERK1/2 and PI3K/AKT pathways. Int J Nanomedicine (2019) 14:441–55. doi: 10.2147/ijn.S188439 PMC6330985 30666106

[31] Liang H Huang J Huang Q Xie YC Liu HZ Wang HB . Pharmacological inhibition of Rac1 exerts a protective role in ischemia/reperfusion-induced renal fibrosis. Biochem Biophys Res Commun (2018) 503:2517–23. doi: 10.1016/j.bbrc.2018.07.009 30208520

[32] Han X Hu J Zhao W Lu H Dai J He Q . Hexapeptide induces M2 macrophage polarization *via* the JAK1/STAT6 pathway to promote angiogenesis in bone repair.[J]. Exp Cell Res (2022) 413:113064. doi: 10.1016/j.yexcr.2022.113064 35167829

[33] Zhang M Liu Zongzhi Z Aoshima K Cai WL Sun H Xu T . CECR2 drives breast cancer metastasis by promoting NF-κB signaling and macrophage-mediated immune suppression.[J]. Sci Transl Med (2022) 14:eabf5473. doi: 10.1126/scitranslmed.abf5473 35108062 PMC9003667

[34] Lee YS Song SJ Hong H Oh BY Lee WY Cho YB . The FBW7-MCL-1 axis is key in M1 and M2 macrophage-related colon cancer cell progression: validating the immunotherapeutic value of targeting PI3Kγ. Exp Mol Med (2020) 52:815–31. doi: 10.1038/s12276-020-0436-7 PMC7272616 32444799

[35] Li Y Li M Wei R Wu J . Identification and functional analysis of EPOR tumor-associated macrophages in human osteosarcoma lung metastasis. [J] .J Immunol Res (2020) 2020:9374240. doi: 10.1155/2020/9374240 32908942 PMC7450330

[36] Ran T Chen Z Zhao L Ran W Fan J Hong S . LAMB1 is related to the T stage and indicates poor prognosis in gastric cancer. Technol Cancer Res Treat (2021) 20:15330338211004944. doi: 10.1177/15330338211004944 33784890 PMC8020091

[37] Hess G Arnold W Meyer BKH . Inhibition of hepatitis b virus deoxyribonucleic acid polymerase by the 5'-triphosphates of 9-beta-D-arabinofuranosyladenine and 1-beta-D-arabinofuranosylcytosine. Antimicrob Agents Chemother (1981) 19:44–50. doi: 10.1128/aac.19.1.44 6166246 PMC181355

[38] Overington JP Al-Lazikani B Hopkins AL . How many drug targets are there? Nat Rev Drug Discovery (2006) 5(12):993–6. doi: 10.1038/nrd2199 17139284

[39] AbdeL-Hafiz H Fatima K Qadri I . Hepatitis b virus X protein impedes the DNA repair *via* its association with transcription factor, TFIIH. BMC Microbiol (2011) 11(1):48. doi: 10.1186/1471-2180-11-48 21375739 PMC3060106

[40] Huang F Wong D Seto W Mak LY Cheung TT Yuen MF . Tumor suppressive role of mitochondrial sirtuin 4 in induction of G2/M cell cycle arrest and apoptosis in hepatitis b virus-related hepatocellular carcinoma. Cell Death Dis (2021) 7(1):88. doi: 10.1038/s41420-021-00470-8 PMC8087836 33931611

[41] Xu X Ye L Zhang Q Shen H Li S Zhang X . Group-2 innate lymphoid cells promote hepatocellular carcinoma progression *via* CXCL2-neutrophil induced immunosuppression. [J]. Hepatol (2021) 74(5):2526–43. doi: 10.1002/hep.31855 PMC8597094 33829508

[42] Zhao Z Wang C Chu P Lu X . Key genes associated with tumor-infiltrating non-regulatory CD4- and CD8-positive T cells in microenvironment of hepatocellular Carcinoma.[J]. Biochem Genet (2022) 60(5):1762–1780. doi:10.1007/s10528-021-10175-3 PMC9470630 35092558

[43] Meng Q Duan X Yang Q Xue D Liu Z Li Y . SLAMF6/Ly108 promotes the development of hepatocellular carcinoma *via* facilitating macrophage M2 polarization. Oncol Lett (2022) 23(3):83–3. doi: 10.3892/ol.2022.13203 PMC8805185 35126725

[44] Qu N Bo X Li B Ma L Wang F Zheng Q . Role of N6-methyladenosine (m^6^A) methylation regulators in hepatocellular carcinoma. Front Oncol (2021) 11:755206–6. doi: 10.3389/fonc.2021.755206 PMC8529104 34692544

[45] Zhou J Zhang A Fan L . HSPA12B secreted by tumor-associated endothelial cells might induce M2 polarization of macrophages *via* activating PI3K/Akt/mTOR Signaling.[J]. OncoTargets Ther (2020) 13:9103–11. doi: 10.2147/ott.S254985 PMC7494226 32982299

[46] Limviphuvadh V Tan CS Konishi F Jenjaroenpun P Xiang JS Kremenska Y . Discovering novel SNPs that are correlated with patient outcome in a Singaporean cancer patient cohort treated with gemcitabine-based chemotherapy. BMC Cancer. (2018) 18(1):555. doi: 10.1186/s12885-018-4471-x 29751792 PMC5948914

[47] Chang J Nie X Chang HE Han Z Taylor J . Transcription of hepatitis delta virus RNA by RNA polymerase II. J Virol (2008) 82(3):1118–27. doi: 10.1128/JVI.01758-07 PMC2224410 18032511

[48] Fan Y Liang Y Liu Y Fan H . PRKDC promotes hepatitis b virus transcription through enhancing the binding of RNA pol II to cccDNA. Cell Death Dis (2022) 13:404. doi: 10.1038/s41419-022-04852-3 35468873 PMC9038722

[49] Cao P Yang A Wang R Xia X Zhai Y Li Y . Germline duplication of SNORA18L5 increases risk for HBV-related hepatocellular carcinoma by altering localization of ribosomal proteins and decreasing levels of p53. Gastroenterology (2018) 155:542–56. doi: 10.1053/j.gastro.2018.04.020 29702115

[50] Zhang Z Wu Q Fang M Liu Y Jiang J Feng Q . HERC3 directly targets RPL23A for ubiquitination degradation and further regulates colorectal cancer proliferation and the cell cycle. Int J Biol Sci (2022) 18:3282–97. doi: 10.7150/ijbs.72014 PMC9134906 35637966

[51] Golfinopoulos V Pentheroudakis G Goussia A Siozopoulou V Bobos M Krikelis D . Intracellular signalling *via* the AKT axis and downstream effectors is active and prognostically significant in cancer of unknown primary (CUP): a study of 100 CUP cases. Ann Oncol (2012) 23:2725–30. doi: 10.1093/annonc/mds097 22565124

[52] Challa S Khulpateea BR Nandu T Camacho CV Ryu KW Chen H . Ribosome ADP-ribosylation inhibits translation and maintains proteostasis in cancers. Cell (2021) 184:4531–4546.e26. doi: 10.1016/j.cell.2021.07.005 34314702 PMC8380725

[53] Xue J Xiao T Wei S Sun J Zou Z Shi M . miR-21-regulated M2 polarization of macrophage is involved in arsenicosis-induced hepatic fibrosis through the activation of hepatic stellate cells. J Cell Physiol (2021) 236:6025–41. doi: 10.1002/jcp.30288 33481270

[54] Wang M Zhang M Fu L Lin J Zhou X Zhou P . Liver-targeted delivery of TSG-6 by calcium phosphate nanoparticles for the management of liver fibrosis. Theranostics (2020) 10:36–49. doi: 10.7150/thno.37301 31903104 PMC6929629

[55] Bility MT Nio K Li F McGivern DR Lemon SM Feeney ER . Chronic hepatitis c infection-induced liver fibrogenesis is associated with M2 macrophage activation. Sci Rep (2016) 6:39520. doi: 10.1038/srep39520 28000758 PMC5175173

