# Corrigendum: Exploring the molecular mechanism of hepatitis virus inducing hepatocellular carcinoma by microarray data and immune infiltrates analysis

**DOI:** 10.3389/fimmu.2023.1168774

**Published:** 2023-02-28

**Authors:** Yong-Zheng Zhang, Amir Zeb, Lu-Feng Cheng

**Affiliations:** Department of Pharmacology, School of Pharmacy, Xinjiang Medical University, Urumqi, China

**Keywords:** hepatitis virus, hepatocellular carcinoma, immune infiltration, viral carcinogenicity, macrophage polarization, computer simulation

In the published article, there was an error in **Figure 6** as published. The corrected **Figure 6** and its caption appear below.

**Figure 6:**
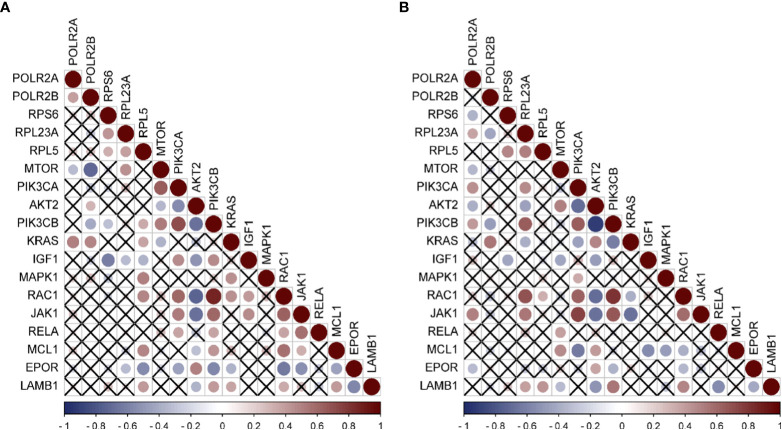
Expression correlation analysis of hub genes and Immune related genes **(A)** Correlation analysis of HBV-HCC immune pathway related genes and hub genes by using GSE55092. **(B)** Correlation analysis of HCV-HCC immune pathway related genes and hub genes by using GSE69715. Red was positively correlated and blue was negatively correlated. The size of the circle represented the magnitude of correlation, and the cross indicated no correlation.

**Figure 6** caption (to be used in the figure only):

Expression correlation analysis of hub genes and Immune related genes **(A)** Correlation analysis of HBV-HCC immune pathway related genes and hub genes by using GSE55092. **(B)** Correlation analysis of HCV-HCC immune pathway related genes and hub genes by using GSE69715. Red was positively correlated and blue was negatively correlated. The size of the circle represented the magnitude of correlation, and the cross indicated no correlation

The authors apologize for this error and state that this does not change the scientific conclusions of the article in any way. The original article has been updated.

